# Draft Genomes of *Anopheles cracens* and *Anopheles maculatus*: Comparison of Simian Malaria and Human Malaria Vectors in Peninsular Malaysia

**DOI:** 10.1371/journal.pone.0157893

**Published:** 2016-06-27

**Authors:** Yee-Ling Lau, Wenn-Chyau Lee, Junhui Chen, Zhen Zhong, Jianbo Jian, Amirah Amir, Fei-Wen Cheong, Jia-Siang Sum, Mun-Yik Fong

**Affiliations:** 1 Department of Parasitology, Faculty of Medicine, University of Malaya, Kuala Lumpur, Malaysia; 2 Singapore Immunology Network (SIgN), Agency for Science, Technology and Research (A*STAR), Singapore, Singapore; 3 Beijing Genomics Institute (BGI), ShenZhen, China; Université Pierre et Marie Curie, FRANCE

## Abstract

*Anopheles cracens* has been incriminated as the vector of human knowlesi malaria in peninsular Malaysia. Besides, it is a good laboratory vector of *Plasmodium falciparum* and *P*. *vivax*. The distribution of *An*. *cracens* overlaps with that of *An*. *maculatus*, the human malaria vector in peninsular Malaysia that seems to be refractory to *P*. *knowlesi* infection in natural settings. Whole genome sequencing was performed on *An*. *cracens* and *An*. *maculatus* collected here. The draft genome of *An*. *cracens* was 395 Mb in size whereas the size of *An*. *maculatus* draft genome was 499 Mb. Comparison with the published Malaysian *An*. *maculatus* genome suggested the *An*. *maculatus* specimen used in this study as a different geographical race. Comparative analyses highlighted the similarities and differences between *An*. *cracens* and *An*. *maculatus*, providing new insights into their biological behavior and characteristics.

## Introduction

Many tropical and subtropical regions of Asia are still endemic to malaria with various species of *Anopheles* mosquitoes acting as vectors [1–3]. On top of that, some of these malaria vectors are involved in transmission of human and zoonotic filariasis in this region [4, 5]. The involvement of *Plasmodium knowlesi* in human malaria transmission has further complicated the malaria transmission profile in Southeast Asia, particularly in Malaysia [6]. Worse still, the vectors responsible for transmission of knowlesi malaria in human population are different from the vectors of other human malaria parasites in some affected areas. Take peninsular Malaysia for example, the vector of *P*. *knowlesi* is *Anopheles cracens*, which can be found at the edge of forests in hilly areas [7]. The vectors of human non-knowlesi malaria parasites in peninsular Malaysia include *An*. *campestris* and *An*. *epiroticus* along the coast, and *An*. *maculatus* in the hilly zones [8].

Despite being regarded as anthropophilic, *An*. *maculatus* has been shown to feed on monkeys when presented with the opportunity [9–11]. Besides, *An*. *maculatus* was shown to be susceptible to simian malaria parasites in laboratory settings [12, 13]. In addition, *An*. *maculatus* and *An*. *cracens* were collected from the same field sites used by a number of previous studies [14–16], implying an overlap of geographical distribution for these two species in peninsular Malaysia. However, unlike *An*. *cracens*, the wild *An*. *maculatus* was found to be negative for *P*. *knowlesi* sporozoites in a field study conducted in peninsular Malaysia [8]. On the other hand, *An*. *cracens*, a simio-anthropophilic mosquito [16], was proven to be a good laboratory vector of *P*. *vivax* and *P*. *falciparum* [17, 18]. As reasons behind the varied susceptibilities of each anopheline to different species of *Plasmodium* spp. in wild and laboratory settings are yet to be completely understood, in-depth studies of these mosquitoes at the genomic level may provide new insights to answer the question. In this report, we presented full draft genomes of *An*. *maculatus* and *An*. *cracens*, the established falciparum malaria and knowlesi malaria vectors in peninsular Malaysia respectively. With these draft genomes, we performed genomic comparisons with the archived genomes of other human malaria vectors [19–23], in an attempt to get a better understanding of these medically important mosquitoes.

## Materials and Methods

### Ethical Approval

This study was approved by Institutional Animal Care and Use Committee of University of Malaya (PAR/19/02/2013/AA) and Ethical and Research Review Committee of the Ministry of Health, Malaysia (NMRR-11-1050-110619).

### Mosquito sample preparation and genomic sequencing

Catchment of *An*. *cracens* and *An*. *maculatus* was conducted in peninsular Malaysia at location points N04°12.584’ E101°52.515’ and N05°45’16.8042” E101°44’48.1914” respectively, based on sites reported by previous studies [15, 24]. Bare leg catch (BLC) and human-baited net trapping methods were used as described previously [25, 26]. The collection was carried out between 18:00 and 23:30 hours. The identity of the mosquito species was assessed using taxonomy morphological keys [9, 27]. Each of the *An*. *cracens* and *An*. *maculatus* collected was kept in separate glass collecting tubes for further processing in laboratory.

High molecular weight genomic DNA was isolated from individual mosquitoes using DNeasy Blood and Tissue Kit (QIAGEN, Germany). The DNA yield was measured spectrophotometrically (*Qubit* fluorometer dsDNA HS Kit, Invitrogen), and the DNA integrity was verified by agarose gel electrophoresis and Bioanalyzer (2100, Agilent). Meanwhile, the identities of the mosquitoes were further verified by PCR directed against the following anopheline genes: mitochondrial cytochrome oxidase I (mtCOI) (primers used: LCO1490: 5’ GGT CAA CAA ATC ATA AAG ATA TTG G 3’ and HCO2198: 5’ TAA ACT TCA GGG TGA CCA AAA AAT CA 3’), mtCOII (primers used: C2-J-3138: 5’-AGA GCT TCT CCT TTA ATG GAA CA-3’ and C2-N-2686: 5’-CAA TTG GTA TAA AAC TAT GAT TTG-3’) and internal transcribed spacer II (ITS II) (primers used: ITS2A: 5’TGTGAACTGCAGGACA3’ and ITS2B: 5’TATGCTTAAATTCAGGGGGT3’) [28–30].

Genomic DNA was sheared into fragments and six libraries were constructed with inserted fragment sizes ranging from 200 bp, 500 bp, 800 bp, 2 kb, 5 kb, and 10 kb by the manufacturer’s library kit (Illumina). In order to produce sufficient amounts of DNA for libraries, 250–500 ng of genomic DNA were subjected to whole-genome amplification using the REPLI-g midi kit (Qiagen). Subsequently the libraries were sequenced using the Illumina-HiSeq^™^ 2000 platform with paired-end sequencing approaches, yielding raw sequence data of 94.89 Gb for *An*. *cracens* and 59.56 Gb for *An*. *maculatus* (S1 Table). Subsequently, artificial reads and low quality paired reads derived mainly from adapter contamination were filtered to facilitate the assembling works. Base-calling duplicates caused by SOLEXA-pipeline were filtered at the threshold of Euclidean distance ≤ 3 and a mismatch rate of ≤ 0.1. The PCR-derived duplicated reads (long inserts of ≥ 2 Kb and short inserts of 150–500 bp) were filtered to ensure good quality scaffold construction. Low quality sequences with “N” base content higher than 10% were removed as well. The data size were reduced to 83.77 Gb for *An*. *cracens* (sequencing depth of 212.09-fold) and 46.53 Gb for *An*. *maculatus* (sequencing depth of 93.21-fold) (S2 Table).

### Genome assembly

Prior to assembly, the sequencing error was corrected based on *k*-mer frequency information to reduce the memory consumption in De Bruijn graph algorithm construction. We had selected 17-mers and corrected the sequencing errors for frequency lower than 4. For *An*. *cracens*, the total bases used were 10.81 Gb. For *An*. *maculatus*, the total bases used were 231.69 Mb. The corrected reads were assembled using SOAP *de novo* [31], which assembles short oligonucleotides into contigs and scaffolds through De Bruijn graph algorithm. Only short insert size (< 1 kb) of single and paired end reads were recruited in the assembly to avoid chimeric reads. Removal of errors such as tips, low coverage linkages and bubbles, and tiny repeats resolving were done. The graphs were transformed into a contig plot by transforming linearly connected *k-mers* into pre-contig node. Bubbles were traced with Dijkstra’s algorithm (Skiena), which were then merged into a single pathway when identical branches were detected. As a result, repetitive sequences collapsed and consensus sequences were sorted.

With PE reads, contigs were linked into a scaffolding graph. Connections between contigs comprised of the branch length (the gap size calculated from the insert size of the PE reads) and the edges of the graph. Subsequently, the interleaving contigs were transformed into a linear structure by applying sub-graph linearization. For repeat contigs, repeat masking was performed to mask the complicated connections. Therefore, contigs in any non-linear structure were considered compatible. Following this, PE reads were applied with increasing insert sizes (from the shortest 170 bp reads to the longest 10 kb reads). The gaps between scaffolds were then filled by aligning the PE reads, retrieving those that had one read that was well-aligned on a contig and another located within the gap region. Local assembly was performed with these retrieved reads. Besides, SSPACE software was applied to generate the scaffolds [32]. The possible contigs were extended with unmapped sequence reads based on the overlap relationships between contigs and reads. Subsequently, the scaffolds were constructed by pre-assembling contigs using PE read data. The genomic GC content was analysed to evaluate nucleotide distribution, the randomness of sequencing, as well as tracing possible sample contamination. Non-overlapping sliding windows with a 10 kb size were used, and the GC content and average depth among the windows was calculated. GC content distribution was determined by using 500 bp bins (with 250 bp overlap) for *An*. *cracens* and 200 bp bins (with 100 bp overlap) for *An*. *maculatus* sliding along the genomes.

### Repeats

A combination of homology-based and *de novo* approaches were used to detect interspersed repeated sequences. For homology-based approach, Repbase, which archives annotated sequences representing repeats from different families were applied. Transposable elements (TEs) were predicted at genomic and proteomic levels. For DNA-level TEs prediction, RepeatMasker was used together with the Repbase library [33]. For protein-level TEs prediction, RepeatProteinMask was applied to perform RMblast2.0 against TE protein database in Repbase [34]. For *de novo* approach, LTR_Finder, PILER and REPEATSCOUT were used [35–37]. Outputs from these softwares were fused into a library, and RepeatMasker was used to identify and categorize the homologous repeats in the draft genomes. Types of TEs, which encompass DNA transposons, long terminal repeat (LTR), short interspersed elements (SINE) and long interspersed elements (LINE) were quantified.

### Gene annotation, orthologous gene clustering and phylogeny

Gene annotation was done using three techniques, namely *de novo* approach, homolog approach, and transcript approach. For *de novo* approach, AUGUSTUS, GlimmerHMM, SNAP and GENSCAN [38–41] were used. False positive results were reduced by filtering genes with coding length longer than 150 bp. For homolog approach, protein sets of *An*. *gambiae*, *An*. *darlingi*, *An*. *sinensis*, *Ae*. *aegypti*, *Cx*. *quinquefasciatus*, *D*. *melanogaster* were recruited for gene prediction of *An*. *cracens*. For gene prediction of *An*. *maculatus*, protein sets of *An*. *gambiae*, *An*. *darlingi*, *Ae*. *aegypti*, *Cx*. *quinquefasciatus* and *D*. *melanogaster* were used in the homolog method. The protein sets were mapped to the assembled genomes using BLAST with E-value of 10^−5^. The most homologous protein for each genomic locus showing multiple matches was selected. Regions with homology lower than 25% of the query protein were removed. A 500 bp-nucleotide sequence was extended at both alignment ends and the gene structures were predicted using program GeneWise2.2.0 [42]. For transcript approach, the reads were aligned through TopHat, followed by assembling with Cufflinks software. Data generated by these approaches were consolidated with software GLEAN 2.2 [43], from which consensus gene sets were generated.

Functional annotation was done based on the best alignment match for individual genes using a number of databases. With InterProScan, the motifs and domains of genes were determined by scanning the sequences against protein databases such as Pfam, SMART, PROSITE, PRINTS and ProDom [44–49]. Gene Ontology (GO) of the genes were collected based on the corresponding InterPro entry [50]. Unique genes for each species under study were identified, where their annotated functions and cellular pathways were studied using Kyoto Encyclopaedia of Genes and Genomes (KEGG) [51]. The function prediction was further complemented with Swiss-Prot and TrEMBL [52].

Apart from analytical and functional analyses, pair-wise whole genome synteny analyses were performed with LASTZ on draft genomes of *An*. *cracens* and *An*. *maculatus* using *D*. *melanogaster* genome as the target genome. Besides, analyses were done on orthologous gene clusters to find out the single copy gene families and multi-gene families, which are conserved among species. A total of 21 members from taxonomic order Diptera were recruited to perform genome-wise comparison with genomes of *An*. *cracens* and *An*. *maculatus*. Syntenic blocks between the genomes were detected with LASTZ. Orthology assignment of the recruited species was then performed. The genes were clustered into gene families. Data from seven species (*An*. *cracens*, *An*. *maculatus*, *An*. *darlingi*, *An*. *gambiae*, *An*. *sinensis*, *Ae*. *aegypti*, and *Cx*. *quinquefasciatus*) were further selected to examine the extent of orthologous group sharing among them. From the gene clustering, single copy families were obtained. Phylogeny was calculated using maximum likelihood analyses of 226 single copy gene families. A phylogenetic tree was constructed on one-fold degenerate sites, using *D*. *melanogaster* as the outgroup.

### Analyses on positively selected genes (PSG)

In the process of evolution, different forms of natural selection happen. Directional selection, which favours the extreme phenotypes of a population, can lead to divergence and speciation. The event induces higher rate of non-synonymous substitution (dN) than synonymous substitution (dS) at orthologous genes. Hence, the extent of this event can be evaluated by investigating the PSGs via dN/dS ratio tests.

Genomes of *An*. *maculatus*, *An*. *cracens*, *An*. *gambiae*, *An*. *darlingi*, and *An*. *sinensis* were recruited. To identify PSGs within the genomes, the single-copy orthologous genes were identified [53]. PRANK alignment program was used to conduct multiple nucleotide alignments for coding DNA sequences of the found orthologous gene set [54]. GBlocks program was applied to eliminate poorly aligned positions and divergent regions of a DNA alignment. Following this, the dN/dS ratio was calculated using codeml package of phylogenetic analysis by maximum likelihood (PAML) [55]. The results were then filtered by setting P ≤ 0.05 as the cut-off point. To further reduce false positive results, only PSGs whose remaining alignments were longer than 60% of the original sequences in at least three out of the five species recruited were selected. After that, functional annotations of the selected PSGs were investigated using InterPro Scan.

### Odorant receptor (OR) analysis

Mosquitoes rely on odorant reception to trace their hosts. The odorant receptor neurons are coded by the odorant receptor (OR) genes [56]. The NCBI archived sequences of OR and its related proteins in fruit fly and mosquitoes were recruited (n = 1,407). Of these full and partial sequences, redundant and very short sequences were excluded, resulting in 189 query sequences. Subsequently, homolog prediction for ORs of *An*. *maculatus*, *An*. *cracens* and *D*. *melanogaster* were conducted and their respective OR gene family size were calculated.

### Data Reporting

This whole genome shotgun project has been deposited at DDBJ/EMBL/GenBank with the BioProject code PRJNA309364, BioSample code SAMN04432142 and BioProject code PRJNA309622, BioSample code SAMN04437154 for *An*. *cracens* and *An*. *maculatus* respectively.

## Results

### Genome assembly and repeat content

**Fig 1:**
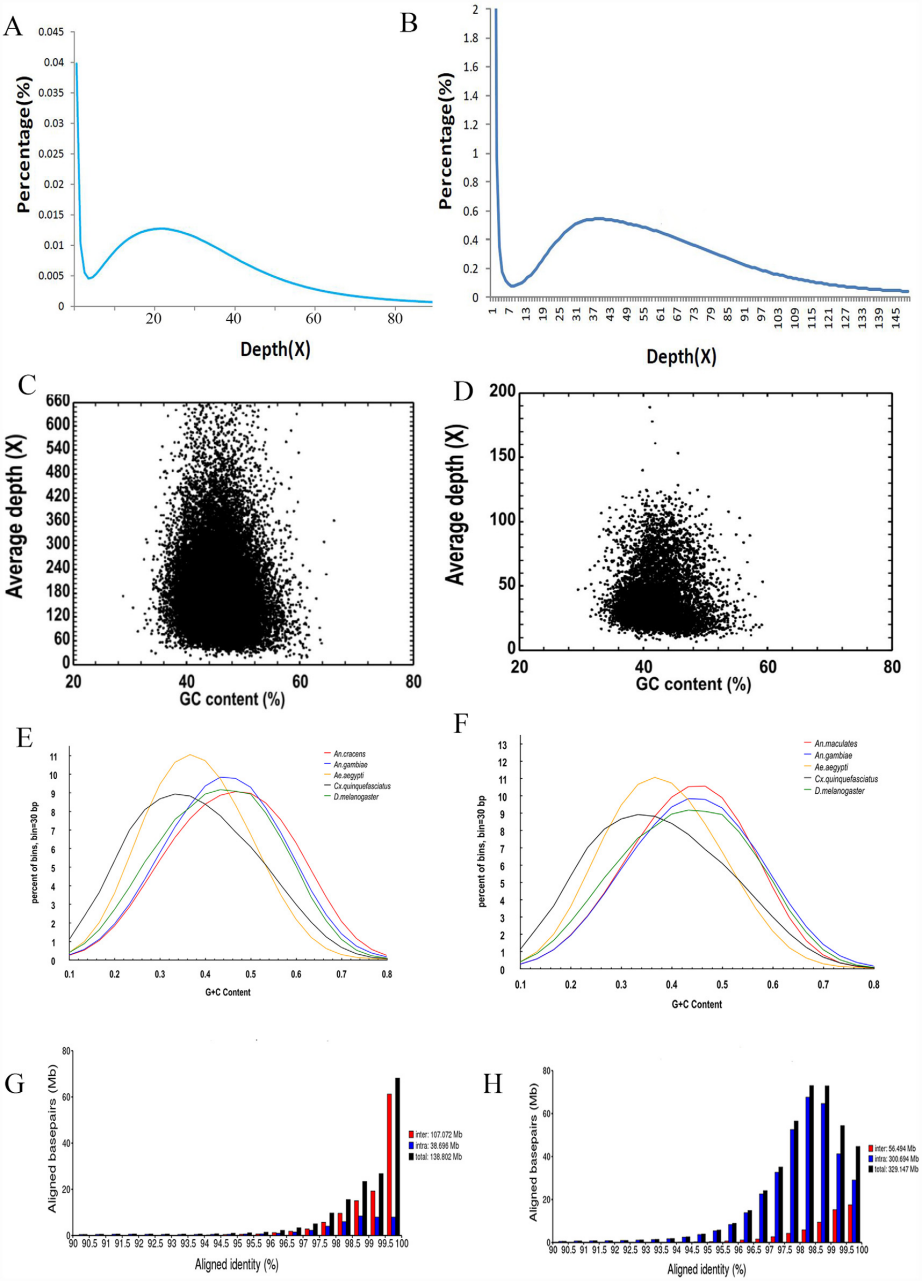
**Genome 17-mer depth distribution for *An*. *cracens* (A) and *An*. *maculatus* (B). The genome GC content of *An*. *cracens* (C) and *An*. *maculatus* (D). The genome GC distribution of *An*. *cracens* (E) and *An*. *maculatus* (F) under comparison with genomes of a few other Diptera members.** For *An*. *cracens*, genomes of *An*. *gambiae*, *Ae*. *aegypti*, *Cx*. *quinquefasciatus* and *D*. *melanogaster* were used for comparison. Five hundred bp bins (with 250 bp overlap) sliding was used. For *An*. *maculatus*, genomes of *An*. *gambiae*, *D*. *melanogaster*, *Apis mellifera*, and *Tribolium castaneum* were used for comparison. Two hundred bp bins (with 100 bp overlap) sliding was used. **Aligned identity distribution of segmental duplication for *An*. *cracens* (G) and *An*. *maculatus* (H).**

Stringent removal of sequencing errors produced draft genomes of smaller sizes than their respective raw sequences (S1 and S2 Tables). The draft genome of *An*. *cracens* was sequenced at 212.09-fold coverage. The total input reads were 108,126,290. The specificity *k*-mer number was 9,082,608,360. The peak of its 17-*mer* distribution was 23, with expected depth of 27.38 according to the distribution curve (Fig 1A). Genome of 5,935 scaffolds totalling 395 Mb in size was generated. No apparent heterozygosis was detected in the genome. The draft genome of *An*. *maculatus* was sequenced at 93.21-fold coverage. The total input reads were 23,168,962. The specificity *k*-mer was 19,461,928,164. The peak of its 17-*mer* distribution was 39 with expected depth of 46.4 according to the distribution curve (Fig 1B). Genome of 10,645 scaffolds totalling 499 Mb was generated (Table 1). The size of *An*. *maculatus* genome was the largest when compared to those of *An*. *cracens* (395 Mb), *An*. *gambiae* (273.1 Mb), *An*. *darlingi* (137 Mb), and *An*. *sinensis* (375.8 Mb). The larger genome sizes yielded for *An*. *maculatus* and *An*. *cracens* could be attributed to the performed sequencing depth. Nevertheless the genomes of *Anopheles* spp. recruited in this study were smaller than those of *Ae*. *aegypti* (1,311 Mb) and *Cx*. *quinquefasciatus* (579 Mb). From sequence analyses, GC contents of *An*. *cracens* and *An*. *maculatus* were found to be 45.7% and 43.3% respectively (Fig 1C and 1D). The values of genome GC content of *An*. *cracens* and *An*. *maculatus* are indeed similar to other *Anopheles* genomes (Table 1), as well as genomes of other Diptera members (Fig 1E and 1F).

**Table 1:** Genome assembly comparison of *An*. *cracens* and *An*. *maculatus* with other mosquitoes. Data on *An*. *cracens* and *An*. *maculatus* were generated from this study whereas the details about other species were obtained from Vectorbase (*An*. *gambiae* GCA_000005575.2, *An*. *darlingi* GCA_000211455.3, *An*. *sinensis* GCA_000472065.2, *Ae*. *aegypti* GCA_000004015.1, *Cx*. *Quinquefasciatus* GCA_000209185.1)

	*An*. *cracens*	*An*. *maculatus*	*An*. *gambiae*	*An*. *darlingi*	*An*. *sinensis*	*Ae*. *aegypti*	*Cx*. *quinquefasciatus*
Version	-	-	AgamP4	AdarC3	AsinS2	AaegL3	CpipJ2
Genome size (Mb)	395	499	273.1	137	375.8	1,311	579
# Contigs	14,791	24,236	16,824	5,683	30,931	36,206	48,671
Contigs N50 (kb)	37.8	29.9	85.6	51.2	18	82.6	28.6
# Scaffolds	5,935	10,645	8	2,221	10,448	4,758	3171
Scaffolds N50 (kb)	151.9	181.3	49,364	115.1	579.1	1,547	486.8
GC (%)	45.7	43.3	44.3	48.2	42.6	38.2	37.4
# Protein-coding genes	18,450	24,460	12,457	10,457	16,766	15,419	18,883
**Non-coding RNA genes**							
# miRNA	92	165	187	105	41	165	134
# tRNA	655	723	450	346	348	995	-
# snRNA	46	70	50	30	-	88	72

Transposable elements (TEs) within the genomes were deciphered. There were repeat contents of 35.07% (equivalent to 132.25 Mb of DNA) within the assembled genome of *An*. *cracens*, encompassing 19.55% LTR, 8.61% LINE, 6.85% DNA transposons, 0.26% SINE, 3.31% unclassified dispersed elements, and less than 0.01% of other repeat contents (S3 Table). The genome of *An*. *maculatus* had 97.44 Mb of TEs (equivalent to 19.81% of the generated draft genome). Among the repeat contents, LINE occupied the highest fraction (7.50%), followed by LTR (7.11%), unclassified dispersed elements (3.47%), and 2.6% were DNA transposons. SINEs contributed to 1.6% of the TEs, and less than 0.01% was considered as other repeat contents (S3 Table). We also investigated segmental duplications of the genomes, and estimated 138.802 Mb of segmental duplication for *An*. *cracens* draft genome (Fig 1G). The *An*. *maculatus* draft genome was estimated to have 329.147 Mb of segmental duplications (Fig 1H).

### Gene annotation

**Fig 2:**
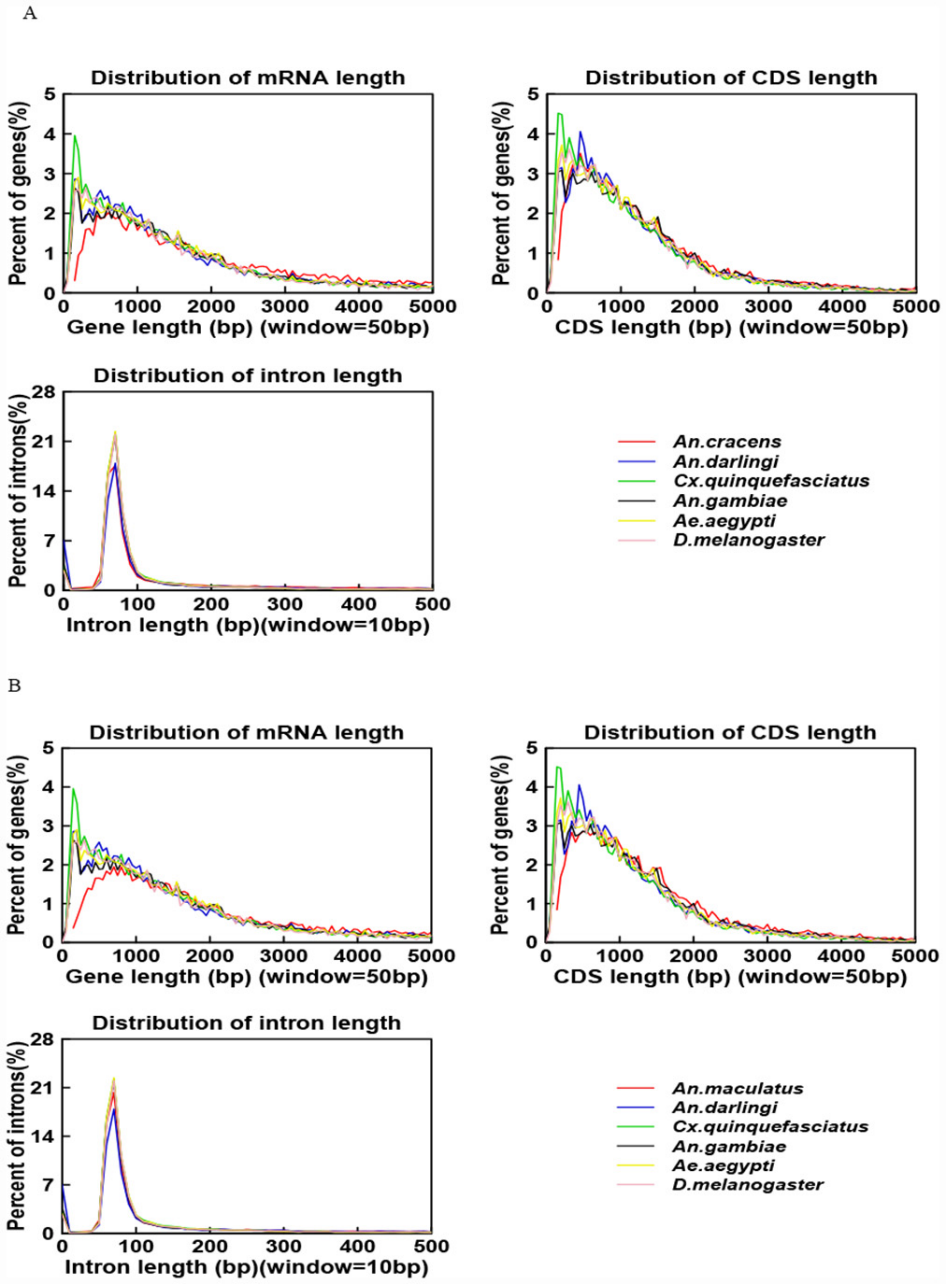
Distribution comparisons of several features in the final gene set to homolog species for *An*. *cracens* (A) and *An*. *maculatus* (B).

From the draft genome of *An*. *cracens*, a total of 18,450 protein coding genes were predicted, giving rise to average transcript length of 3,577.44 bp and average coding DNA sequence (CDS) length of 1,479.47 bp. Overall, the genome of *An*. *cracens* is predicted to harbour an average of 4.07 exons per gene, with the average exon length of 363.75 bp and average intron length of 683.98 bp (S4 Table). On the other hand, draft genome of *An*. *maculatus* was predicted to harbour 24,460 protein-coding genes, which bring about an average transcript length of 4304.06 bp and average CDS length of 1,561.13 bp. On average, there were 4.2 exons per gene, with average exon length of 371.67 bp. The average intron length of the genome was predicted to be 857.08 bp (S4 Table). When compared against recruited species in homology-based annotation, *An*. *cracens* (Fig 2A) and *An*. *maculatus* (Fig 2B) showed close resemblance to other *Anopheles* spp. than non-anopheline culicines and *D*. *melanogaster* in distribution pattern of several annotation features, particularly on distribution of intron length. The tight and narrow peaks seen in the plots of intron length distribution indicate relatively compact genomes for these members of Diptera, as compared to genomes of mammals like humans and rats [57].

Five protein databases (InterPro, GO, Swiss-Prot, KEGG, TrEMBL) were used for functional annotation. Of the 18,450 coding genes predicted in *An*. *cracens* genome, 524 genes (2.84%) were unannotated. 13,008 genes (70.50%) were annotated by InterPro, 10,109 genes (54.79%) were annotated by GO, 11,363 genes (61.59%) were annotated through KEGG database, Swiss-Prot annotated 13,337 genes (72.29%), and TrEMBL annotated 17,903 genes (97.04%) (S5 Table). For *An*. *maculatus*, 808 out of 24,460 predicted coding genes (3.30%) were unannotated. A total of 18,117 genes (74.07%) were annotated by InterPro, 14,175 genes (57.95%) were annotated by GO, and 15,598 genes (63.77%) were annotated through KEGG database. Swiss-Prot annotated 18,499 genes (88.05%) and 21,538 genes (88.05%) were annotated with TrEMBL (S5 Table).

Non-coding RNAs (ncRNAs), which are the RNAs not translated into proteins, were characterized. Four types of ncRNAs were annotated in our study, namely microRNA (miRNA), transfer RNA (tRNA), ribosomal RNA (rRNA) and small nuclear RNA (snRNA). In the draft genome of *An*. *cracens*, we found 92 copies of miRNA, with a total length of 8,205 bp, constituting approximately 0.002% of the whole genome. The average length of miRNA was found to be 89.18 bp. Additionally, 655 copies of tRNA were found as well, totalling 50,416 bp (~0.013% of the genome), with an average length of 76.97 bp for each tRNA. A total of 543 copies of rRNA (total length 39,578 bp) were predicted. Of these, 399 copies were annotated as 18S rRNA, 79 copies were 28S rRNA, 19 copies were 5.8S rRNA, and 46 copies were predicted to be 5S rRNA. Besides, a total of 46 copies of snRNA were found, constituting 6,765 bp, equivalent to approximately 0.0018% of the whole genome. Of these snRNAs, 5 copies were predicted to be CD-box snRNAs and the rest were annotated as splicing snRNAs (S6 Table).

For the genome of *An*. *maculatus*, 165 copies of miRNA totalling 15,602 bp (0.0032% of the whole genome) were annotated. The average length of miRNA was 94.56 bp. A total of 723 tRNAs were annotated, totalling 56,246 bp in length (0.0114% of the whole genome). The average length of tRNA was predicted to be 77.08 bp. Besides, the genome of *An*. *maculatus* was found to harbour rRNAs of nearly five times more than that of *An*. *cracens*. A total of 2,601 rRNAs were found, totalling 173,377 bp in length (approximately 0.035% of the whole genome). Of these, 1,865 copies were annotated as 18S rRNA, 504 copies were 28S RNAs, 157 copies were 5.8S rRNAs, and 75 copies were annotated as 5S RNAs. In addition, 70 snRNAs were annotated as well, with total length of 10,256 bp (0.0021% of the whole genome). Of these snRNAs, 12 were annotated to CD-box snRNAs whereas the remaining 58 copies were annotated as splicing snRNAs (S6 Table).

**Fig 3:**
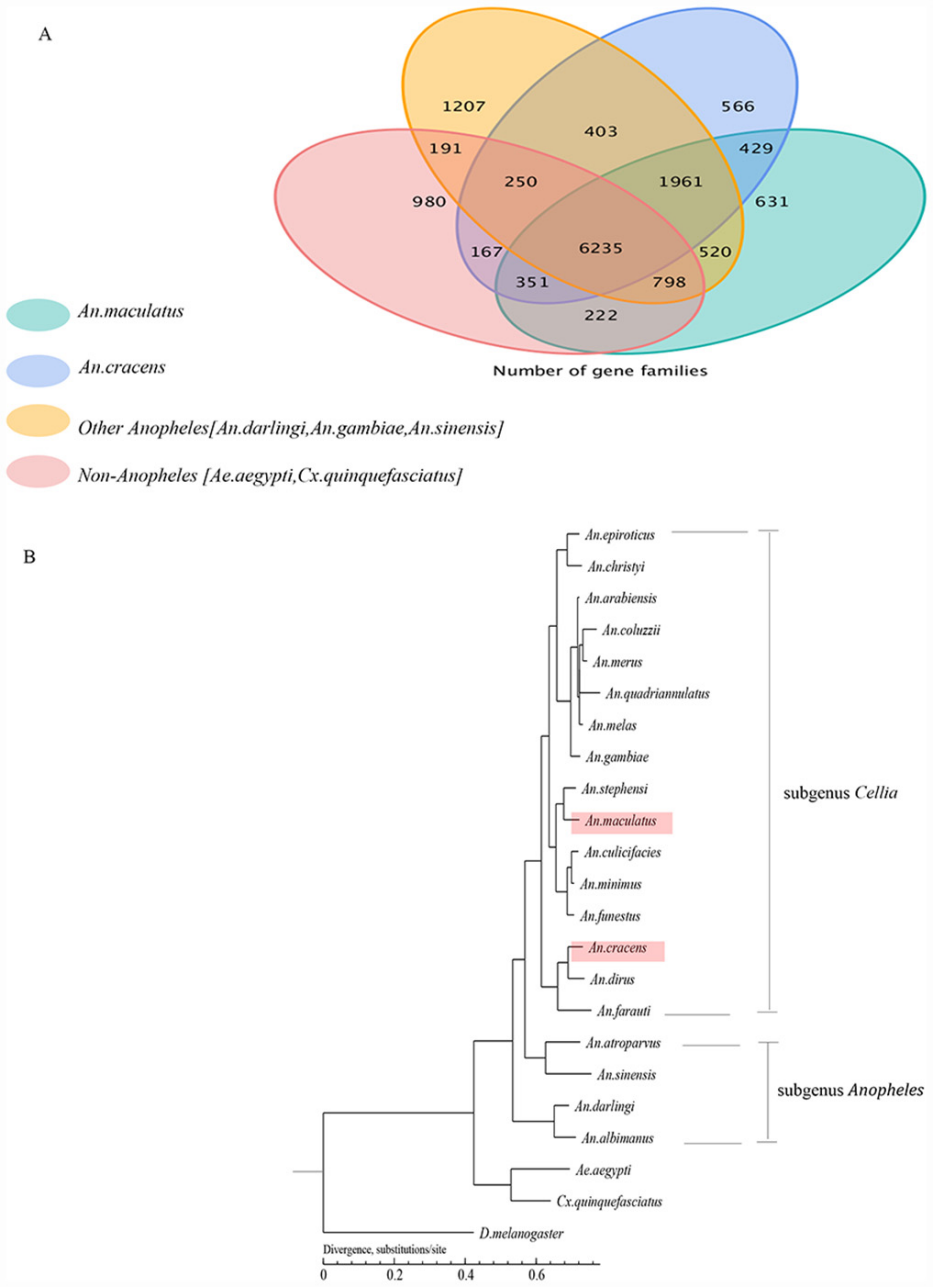
**Venn diagram showing distribution of shared and unique orthologous gene families across species under comparison (A).** Unclustered genes were not included. **Phylogenetic tree constructed with orthologous genes on 1-fold degenerate sites (B).** The branch represents neutral divergence rate.

Based on orthologous gene cluster analyses, the 18,450 protein-coding genes of *An*. *cracens* were clustered into 10,362 families, of which 105 gene families were considered unique to the species. Meanwhile, 1,288 genes did not belong to any gene family due to their uniqueness to *An*. *cracens*. On average, there were 1.66 genes per gene family within *An*. *cracens* genome (S7 Table). For *An*. *maculatus*, the 24,459 predicted protein-coding genes of the generated draft genome were clustered into 11,147 gene families, of which 133 gene families were considered as unique gene families to the species. There were 907 genes which remain unclustered due to their uniqueness to *An*. *maculatus*. On average, there were 2.11 genes per gene family within *An*. *maculatus* genome (S7 Table). By examining the extent of orthologous group sharing among *An*. *cracens*, *An*. *maculatus*, *An*. *darlingi*, *An*. *gambiae*, *An*. *sinensis*, *Ae*. *aegypti* and *Cx*. *quinquefasciatus*, we found that 6,235 gene families were shared across these culicines, and 2,884 gene families were exclusively shared among the *Anopheles* spp. Besides, 429 gene families were exclusively shared between *An*. *cracens* and *An*. *maculatus*. There were 631 gene families were unique to *An*. *maculatus* whereas *An*. *cracens* had 566 unique gene families (Fig 3A). By using 226 single copy gene families, a phylogenetic tree was built (Fig 3B). The phylogenetic tree segregated *D*. *melanogaster* (outgroup) from other culicines. This was followed by another huge segregation of non-*Anopheles* (*Ae*. *aegypti* and *Cx*. *quinquefasciatus*) from the *Anopheles* spp. The tree further segregated the recruited anophelines into two of the seven *Anopheles* subgenera, the subgenus *Anopheles* (the older, more primitive and worldwide distributed subgenus) and subgenus *Cellia* (evolutionarily later, not available in the New World).

### PSG analyses

**Fig 4:**
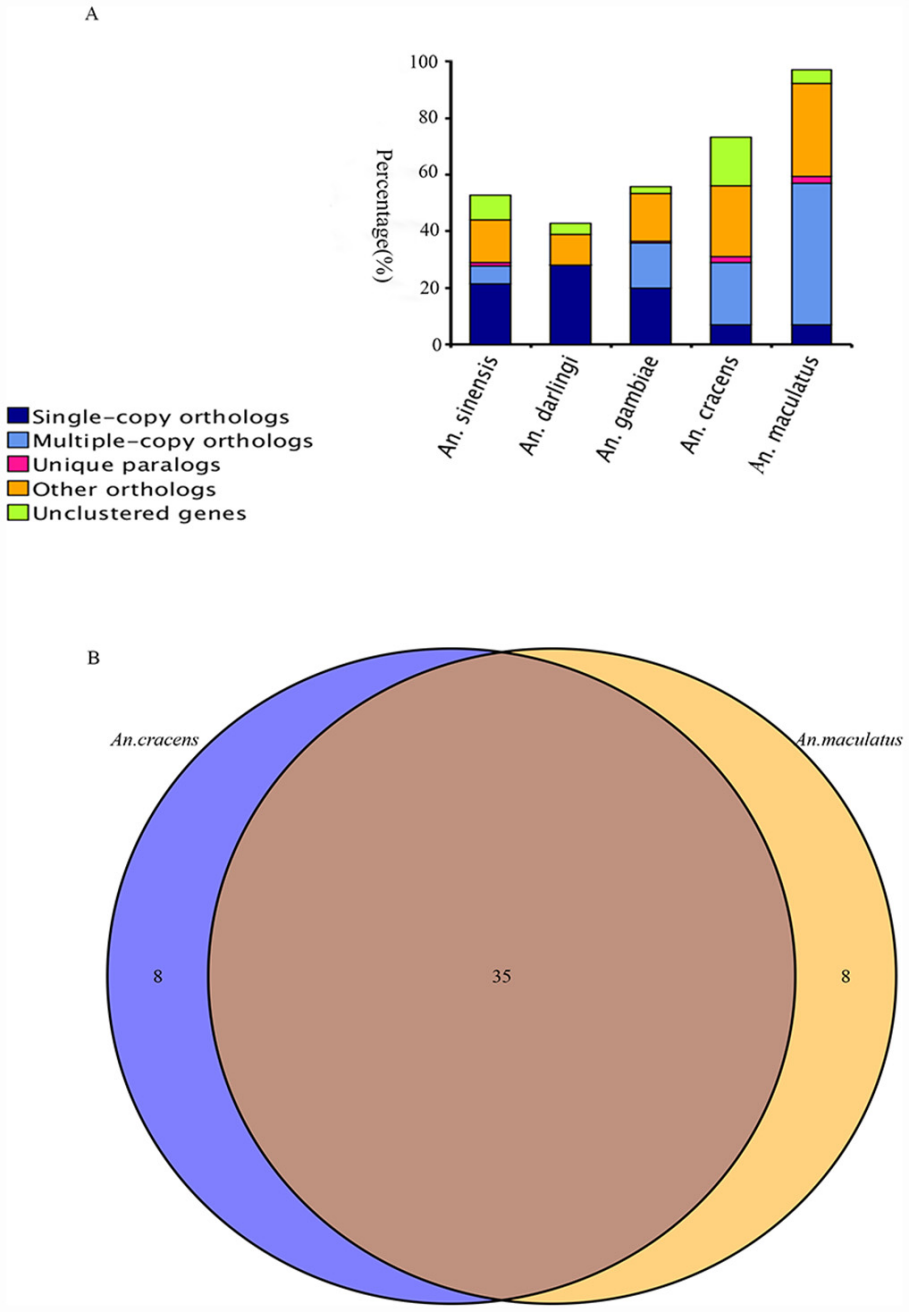
Distribution of orthologs, paralogs and unclustered genes among *An*. *sinensis*, *An*. *darlingi*, *An*. *gambiae*, *An*. *cracens* and *An*. *maculatus* (A). Venn diagram showing positively selected genes (PSGs) shared between *An*. *cracens* and *An*. *maculatus* (B).

A total of 1,021 single copy gene families were found from analyses performed on *An*. *cracens*, *An*. *maculatus*, *An*. *gambiae*, *An*. *darlingi*, and *An*. *sinensis* using TreeFam program (Fig 4A). Following this, 79 PSGs were found for *An*. *cracens* and 40 PSGs were unravelled in *An*. *maculatus* genome (S8 Table). The PSGs of *An*. *cracens* were annotated to 72 protein/ protein domains with identifiable functions including metabolism (28 annotations), gene expression regulation (20 annotations), cellular processes (9 annotations), signal transduction (4 annotations) and organismal system-related functions (4 annotations) (S9 Table). The PSGs of *An*. *maculatus* were annotated to 43 protein/ protein domains with identifiable functions, encompassing metabolic functions (16 annotations), gene expression regulation (15 annotations), cellular processes (3 annotations), signal transduction (2 annotations) and organismal system-related functions (4 annotations) (S10 Table). PSG is related to divergence and speciation process. However, for *An*. *cracens* and *An*. *maculatus*, none of their PSGs found in this study were considered as “unique gene” to the respective species.

### Odorant receptor (OR) genes

By referring to NCBI database, a total of 43 OR genes were predicted for *An*. *cracens*, consisting of 87 gene copies within the genome (S11 Table). For *An*. *maculatus*, 43 OR genes were predicted, encompassing 145 gene copies within its genome (S12 Table). There were 35 OR genes shared between *An*. *cracens* and *An*. *maculatus* (Fig 4B). Under the comparison, eight of the predicted OR genes in *An*. *cracens* were not found in *An*. *maculatus* genome, whereas *An*. *maculatus* had eight OR genes that were not available in *An*. *cracens* genome as well (Fig 4B).

The OR genes that were available in *An*. *cracens* but not in *An*. *maculatus* consisted of nine gene copies. One gene copy was annotated to chromosome region maintenance 1 (CRM1) protein. Five gene copies were annotated to olfactory receptor. One was annotated to sensory neuron membrane protein 1 (SNMP1) and one was annotated to Swiss Cheese (SWS) Isoform A (S13 Table). The eight *An*. *maculatus* OR genes that were not available in *An*. *cracens* consisted of 11 gene copies. One was annotated to Swiss Cheese Isoform C and one was annotated to an odorant reception coreceptor. Three gene copies were annotated to gustatory and odorant receptor 24 (GPRgr24). The remaining six were annotated to olfactory receptor (S14 Table).

### Unique genes with annotation

Of the estimated 1,462 genes unique to *An*. *cracens*, 326 were annotated to functions and mapped to cellular pathways via KEGG database (S15 Table). Most of these genes were mapped to a number of functions across different KEGG classes. Among these 326 unique genes, 96 genes were mapped to pathways related to environmental information processing (signal transduction and membrane transport). There were 90 *An*. *cracens* unique genes annotated to metabolic functions. Besides, 74 of these unique genes were annotated to functions related to cellular processes. A total of 115 genes were annotated to functions related to genetic information processing (i.e. gene expression). A total of 57 genes were annotated to functions related organismal systems.

For *An*. *maculatus*, 358 out of the predicted 1,188 unique genes were annotated by referring to KEGG database (S16 Table). Similar to *An*. *cracens*, most of the annotated genes were mapped to multiple functions across different KEGG classes. There were 102 genes annotated to functions related to environmental information processing. A total of 102 genes were annotated to metabolic functions, 68 unique genes were mapped to cellular processes and 151 genes were linked to genetic information processing. In addition, there were 73 genes annotated to functions related to organismal systems. Interestingly, for both species, the organismal system-related annotations revolved around development of vital organ systems, notably nerve conduction, circadian rhythm, oogenesis and immune system.

## Discussion

### Genome assembly

Despite being smaller than the genomes of *Ae*. *aegypti* [58] and *Cx*. *quinquefasciatus* [59], *An*. *maculatus* draft genome obtained in this study is the largest of the currently available anopheline draft genomes, with the highest number of coding genes. Of note, the genome of *An*. *maculatus* was predicted to have higher repeat rate. This is probably one of the reasons for the much larger genome size for *An*. *maculatus* apart from the high sequencing depth used in this study. Indeed, high repeat rate was also found in the large draft genome of *Ae*. *aegypti* [60]. The content of non-coding RNA genes in *An*. *maculatus* was also higher. For instance, *An*. *maculatus* had the highest number of tRNA and snRNA gene copies upon comparison with *An*. *cracens*, *An*. *gambiae*, *An*. *darlingi*, and *An*. *sinensis*. In addition, *An*. *maculatus* also carried more miRNAs than *An*. *cracens* (165 copies and 92 copies respectively), but fewer than that of *An*. *gambiae* (187 copies). Interestingly, miRNAs were found to be involved in anopheline immune defense against *Plasmodium* oocyst development [61]. It would be interesting to conduct further studies on the interactions between *An*. *maculatus* miRNAs and the infecting *Plasmodium* gametocytes.

Of note, the archived genome of *An*. *maculatus* (BioProject code PRJNA67221, BioSample code SAMN01087922), a specimen from peninsular Malaysia [23], is much smaller than that assembled in this study (144 Mb vs. 499 Mb). This may be due to several factors. Firstly, the difference in insert library preparation between the two studies may contribute to the different assembly outcomes. In this study, size insert libraries of different insert sizes were constructed from DNA of one mosquito. For the previously archived *An*. *maculatus* genome, the small and medium insert libraries were from one individual mosquito whereas the large insert library was from the pooled mosquitoes. Usage of pooled sample for genome sequencing is an acceptable and valuable approach. However, it yields high rates of error that must be corrected. Such corrections result in loss of large amount of data. Besides, the lower sequencing quality by the previous work on *An*. *maculatus* genome (number of scaffolds as 47,797 with N50 of only 4kb) (S17 Table) may be another contributing factor to the difference in genome sizes yielded by these studies. Importantly, we also aligned the previously published *An*. *maculatus* genome against the *An*. *maculatus* draft genome assembled from this study using LASTZ software to evaluate the genome coverage. By setting the *An*. *maculatus* genome from this study as the target genome and using the published *An*. *maculatus* genome as the query genome, we obtained coverage of 99.18% (S18 Table). In addition, we performed *k-mer* analysis on the published *An*. *maculatus* genome by using k = 17. The peak of the 17-mer distribution was 75, with the total *k-mer* count of 49,466,999,758, yielding estimated genome size of 659.56 Mb (S19 Table). A heterozygous peak was seen from the *k-mer* plot at ½ of main peak. Thus, we deduced that the published genome may have heterozygosis. The *k-mer* analysis and genome size estimation for the *An*. *maculatus* genome assembled from this study were elaborated in results section (Fig 1B). To summarize, *k-mer* analysis estimated the *An*. *maculatus* genome sequenced in this study to be 499 Mb. Via 93.12-fold sequencing and assembly with SOAP *de novo* software, we assembled draft genome of 491.84 Mb with effective bases of 470,305,742 and gap length of around 20 Kb. This fine map genome covered 99.18% of the published *An*. *maculatus* genome sequences. Based on the analytical comparisons, the previously published *An*. *maculatus* genome and the *An*. *maculatus* genome assembled in this study are very likely geographical races of *An*. *maculatus* in peninsular Malaysia. Indeed, the *An*. *maculatus* specimens provided for study of Neafsey *et al*. were colonies originated from Jeram Kedah (N02° 54.33' E101° 57.46') of Negeri Sembilan state in west coast of peninsular Malaysia (Daniel Neafsey and Lee HanLim, personal communications) whereas the *An*. *maculatus* specimen used in this study was collected from Jeli (N05°45’16.8042” E101°44’48.1914”) of Kelantan state in the east coast of peninsular Malaysia, a place bordering Thailand. Both locations are more than 300 km apart with a number of mountain ranges in between, segregating the population pools of *An*. *maculatus*. The geographical isolation impedes gene flows and interactions between the two populations, which results in independent divergence of these *An*. *maculatus* populations. With the availability of archived genomes, such small but notable differences can be detected. It would be interesting to study the cross-mating compatibility between these *An*. *maculatus* populations.

### Phylogenetic analyses

In terms of evolutionary study, the phylogenetic tree constructed from this study fits well with the subgenus classification of the recruited mosquitoes. The much older *Anopheles Anopheles* subgenus shared a closer evolutionary relationship with *Ae*. *aegypti* and *Cx*. *quinquefasciatus* when compared with the evolutionarily younger *Anopheles Cellia* subgenus. Indeed, the phylogeny tree constructed in this study was similar to the phylogenetic analysis by Neafsey *et al*. that recruited 18 species of anophelines across the world [23]. Despite that, the difference between the two phylogeny trees was the branching that deciphered evolutionary history of *An*. *gambiae* complex. This may be due to several reasons, such as the recruitment of *An*. *coluzzii* in our phylogenetic analysis, and different recruitment strategies of orthologous gene families into the analyses. In this study, the single copy orthologous gene families were selected based on comparisons of seven species, i.e. *An*. *cracens* (knowlesi malaria vector in peninsular Malaysia), *An*. *maculatus* (falciparum malaria vector in peninsular Malaysia), *An*. *darlingi* (malaria vector in the Neotropics), *An*. *gambiae* (malaria vector in Africa), *An*. *sinensis* (vivax malaria and lymphatic filariasis vector in many parts of Asia), *Ae*. *aegypti* and *Cx*. *quinquefasciatus*. For all that, the overall trend of our phylogenetic analysis was still in good agreement with the previous study.

### PSG analyses

We also looked into the PSGs of *An*. *cracens* and *An*. *maculatus*. For both species, metabolism-related functions occupied the largest fraction of their PSGs, followed by functions related to genetic information processing. Indeed, a previous study on the genome of wild silkworm also revealed metabolism-related annotations as the largest portion of its PSGs [62]. Evolutionary forces may select traits with superior adaptability to the environment, which lead to divergence and speciation in the long run. The PSGs found in the genomes of both species under study were not the unique “branding” genes for the respective species. Nevertheless, these genes contributed critical and positive roles in their evolutionary ancestors’ survival. Eventually they were driven towards speciation, forming the species that we studied in this project.

### OR gene analyses

Odorant receptors are the frontline players in detecting and reacting to scent molecules. Host seeking behavior of mosquitoes is achieved via odor sensing [63]. Each scent particle can stimulate several groups of odorant receptor to varied intensity, and each odorant receptor can react to several scent molecules with different intensity [64, 65]. Hence, the odor response profile of a mosquito determines its host preference range. Based on the OR gene analyses, we found that *An*. *cracens* and *An*. *maculatus* shared most of their OR genes (81.4% of total OR genes found for respective species). The shared OR genes are likely to play vital roles in recruitment of humans as the biting targets by *An*. *cracens* and *An*. *maculatus*. Among these shared OR genes, 10 were annotated to olfactory receptors, which are the rhodopsin-like receptors belonging to the G protein-couple receptor family [66]. The sharing of genes coding for olfactory receptors indicates that both species have olfactory receptors that sense and respond to particular group of smell particles in similar manner. There were five genes annotated to other G protein-coupled receptors including dopamine receptors and tyramine receptors, which have been suggested as the targeted genes for pest control [67–69]. Besides, there were six genes annotated to glutamate receptors including the ionotropic glutamate receptors (IRs), namely IR64a, IR8a, IR25a, and IR76b, which detect chemical stimuli and mediate the sensory perception of smell [70, 71]. Hence, they determine the olfactory behavior of an organism towards a particular smell [72]. Of note, IR64a responds to acidic smells and triggers the acid-avoidance behavior in *D*. *melanogaster* [73]. IR25a was found to be important for temperature-dependent circadian rhythm regulation [74]. Apart from these, three genes were annotated to integrins, namely Integrin α (PS2) and Integrin β (PS/ mys), which are related to smell perception as well [75]. Other annotations found were transient receptor potential channel 1, cyclic nucleotide gated ion channel (CNG), guanine nucleotide binding protein G subunit alpha, teneurin, sensory neuron membrane protein, muscarinic acetylcholine receptor, inositol trisphosphate receptor, gustatory and odorant receptor 22 (GPRgr22) and inhibitory POU protein (I-POU/ acj6). These proteins play important roles in sensory transduction. Of note, GPRgr22 mediates substrate-dependent acceptance/ avoidance behavior of insects [76]. GPRgr22 and GPRgr24 are sufficient to mediate olfactory carbon dioxide chemosensation, which is a mechanism applied by mosquitoes to trace their hosts. I-POU is involved in olfactory behavior regulation such as the chemosensory jump behavior, which is a sudden, upward reflex-like movement off the resting surface upon detection of certain chemicals [77, 78].

Only few OR genes from each species were not shared between *An*. *cracens* and *An*. *maculatus*. In *An*. *cracens*, the additional five non-shared OR genes coding for olfactory receptor may be responsible for the “monkey-seeking” biting behavior that is only occasionaly seen among *An*. *maculatus* from peninsular Malaysia [14]. These five OR genes may widen the biting preference of *An*. *cracens* to monkeys in addition to humans (which may be determined by the 10 OR genes shared with *An*. *maculatus*). Interestingly, *An*. *cracens* from this area was shown to have a monkey to human biting preference ratio of 1:2 [14]. Another non-shared *An*. *cracens* OR gene was coding for sensory neuron membrane protein 1 (SNMP1), which is related to general and pheromone chemoreception [79].

The generated draft genome of *An*. *maculatus* was found to carry six non-shared OR gene copies coding for olfactory receptors. These genes may be responsible for sensing humans and other non-primate animals preferred by *An*. *maculatus* [9, 80]. When other non-primate animals (like cattle) are not available, the odorant receptors of *An*. *maculatus* may become highly sensitive and specific to humans. Of note, two of these five genes (NCBI ID gi.167882457 and gi.167876942) had gene copies that were unique to *An*. *maculatus* (anopheles.maculatus_GLEAN_10016930 and 10000856). Another *An*. *maculatus* OR gene that was not available in the *An*. *cracens* genome was annotated to GPRgr24. As mentioned earlier, GPRgr22 and GPRgr24 are important for olfactory carbon dioxide chemosensation in mosquitoes and the gene coding for GPRgr22 is shared between *An*. *cracens* and *An*. *maculatus*.

### Unique gene annotation

#### Genetic information processing

Our analyses and interpretations on unique genes showed that annotations related to genetic information processing predominate in both *An*. *cracens* (35.28% of unique genes with annotations) and *An*. *maculatus* (42.18% of unique genes with annotations). The annotations revolved around regulation of transcription, translation, post translational processing and DNA repair machinery. Interestingly, high proportion of unique genes annotated to this functional class (73.9% for *An*. *cracens*, 76.8% for *An*. *maculatus*) were coded with functions specific only to regulation of gene expression.

#### Metabolic functions

There were many unique genes from both species with annotations related to metabolic processes as well. Annotations from this KEGG class revolved around protein kinases, metabolism of carbohydrates, proteins, amino acids, lipids, glycans, nucleotides, and organic supplements. Interestingly, *An*. *maculatus* carried a number of unique genes (anopheles.maculatus_GLEAN_10017540, 10013129, and 10008095) annotated to metabolism of polycyclic aromatic hydrocarbons and bisphenol compounds. Such functions may be selected due to the habitat nature of *An*. *maculatus*, which is usually closer to human dwellings [81]. This suggests that human-derived factors may act as selecting pressures on the evolution of mosquitoes.

#### Environmental information processing

Many annotations belonging to environmental information processing, cellular processes and organismal systems are interrelated and a gene may be annotated to closely related functions from these classes. For the unique genes annotated to functions in environmental information processing, most of the genes were annotated to functions associated with signal transduction (85.4% for *An*. *cracens* and 92.2% for *An*. *maculatus*). Annotations for various signal transduction pathways were predicted. These encompassed Wnt signaling, neuroactive ligand-receptor pathways, mTOR signaling, JAK-STAT cascade, MAPK cascade, TGF β signaling, Hedgehog signaling, phosphatidylinositol pathway, and calcium signaling pathways. Interestingly, many genes of this category (11 for *An*. *cracens*, and 14 for *An*. *maculatus*) were annotated to G protein-coupled receptor. Importantly for *An*. *maculatus*, two of these genes (anopheles.maculatus_GLEAN_10016930 and 10000856) were found to be the unique OR genes coding for olfactory receptors as mentioned earlier. In general, these annotations enable the mosquitoes to interact with various factors in their environment such as the temperature, light, humidity, pressure, air movement, host body odor and harmful chemicals. Besides, many of these annotated signaling pathways are also associated with immune response cascades of mosquitoes [82].

#### Cellular processes

A number of annotations to cellular processes are involved in regulation of cell movements, cell growth and cell death. Interestingly, two unique *An*. *cracens* genes (anopheles.cracens_GLEAN_100026190, and 10005166) and four unique *An*. *maculatus* genes (anopheles.maculatus_GLEAN_10002729, 10007372, 10014203, and 10023933) were annotated to cellular functions related to oogenesis. These genes may play important roles in conferring unique features to the gonotrophic cycle, oviposition frequency, survival rates and vectorial capacity of respective species. The gonotrophic cycle for *An*. *cracens* was reported to be 3–5 days [24], whereas the gonotrophic cycle for *An*. *maculatus* was reported as 2.35 days [83]. Gonotrophic cycle characterization is an important way to estimate survival rate of a blood-sucking arthropod. When done correctly, this method yields an accurate estimation of the survival rate of the arthropod under study [84, 85], which is an important factor that determines the vectorial capacity of a mosquito.

#### Organismal system-immune system

For both *An*. *cracens* and *An*. *maculatus*, a large portion of the unique annotations in organismal systems were related to immune system regulation. The arthropods lack an adaptive immune system but pose well-established innate immune system. The innate immune system of insects consists of humoral and cellular mechanisms, with hemocytes as the key players in the immune system [86]. Of note, five unique genes from *An*. *cracens* (anopheles.cracens_GLEAN_10026190, 10026486, 10026487, 10025709, and 10025756) and four from *An*. *maculatus* (anopheles.maculatus_GLEAN_10004760, 10018118, 10014203, and 10023933) were found to be annotated to Toll-like receptor signaling pathway. Toll-like receptors are pattern recognition receptors (PRR) that identify molecules associated with pathogens, constituting the innate immune system of coelomates [87]. The Toll signaling pathway has been reported to play important regulatory roles in anopheline anti-*Plasmodium* immunity [82, 88]. There were two genes from *An*. *cracens* (anopheles.cracens_GLEAN_10026190, 10008293) and seven genes from *An*. *maculatus* (anopheles.maculatus_GLEAN_10004760, 10018118, 10006574, 10001471, 10014203, 10023933, and 10015228) being annotated to functions related to phagocytosis regulation. In mosquitoes, phagocytosis is mediated by hemocytes as well.

There were unique genes annotated to functions orthologous to leukocytes trans-endothelial migration. Within insects, there are two forms of trans-epithelial hemocyte migrations, namely the developmental hemocyte migration during embryogenesis and the sudden onset, wound-induced hemocyte migration, which is dependent on mTOR/P13K signaling [89]. Interestingly, of the four *An*. *cracens* genes annotated to this function (anopheles.cracens_GLEAN_10002582, 10002581, 10023751, 10026190), one gene (anopheles.cracens_GLEAN_10026190) was found to be annotated to mTOR signaling as well. For *An*. *maculatus*, six unique genes were annotated to this cross-barrier migration function (anopheles.maculatus_GLEAN_10020625, 10004760, 10026617, 10024303, 10018118, and 10005396).

Besides, one unique gene from *An*. *cracens* was mapped to functions related to complement pathways and blood coagulation (anopheles.cracens_GLEAN_10006816). Coagulation of hemolymph is a type of humoral immune response in mosquito upon recognition of invading substances [86, 88]. Another type of mosquito humoral immune response is the melanization of hemolymph. One unique gene from *An*. *cracens* (anopheles.cracens_GLEAN_10004497) and nine unique genes from *An*. *maculatus* (anopheles.maculatus_GLEAN_10002239, 10027496, 10014203 10027356, 10008829, 10024562, 10004938, 10014203, and 10023933) were annotated to melanogenesis. Melanization has been reported as an anti-parasitic defense mechanism by mosquitoes [90, 91]. As mentioned earlier, non-coding genes (miRNAs) have been found to play roles in immune defense of anopheline against *Plasmodium* oocyst development [61]. Collectively, these genes constitute the immune defense of mosquitoes.

Although mosquitoes have an established innate immune system, pathogens may come out with evasion mechanisms to infect and colonize the mosquitoes successfully. For instance, vector specific haplotypes of *P*. *falciparum* Pfs47 were shown to mask the parasites from recognition by the immune system of the specific vector [92]. In addition, coinfections with other pathogens or microbiota may change the vectorial competency of a mosquito [93, 94]. These, along with anopheline feeding preferences, may contribute to their varied susceptibilities to all medically important *Plasmodium* spp in natural settings. On the other hand, laboratory settings provide a controlled living condition to the caged mosquitoes, from the type of feeding hosts to the parasite density of the infected blood source used for feeding. A mosquito that is normally, naturally refractory to a parasite may become susceptible to the parasite if the load of “parasite inoculation” is much higher than the normal “dose”, as exemplified by a study on *An*. *quadrimaculatus* and subperiodic *Brugia malayi* [91]. The immune system of the mosquito is overwhelmed and fatigued by the much higher load of parasite invasion, which results in successful invasion and development of some of the inoculated parasites. This may explain the susceptibility of *An*. *maculatus* to simian malaria parasites in laboratory settings [12, 13], as well as the status of *An*. *cracens* as a good laboratory vector for *P*. *vivax* and *P*. *falciparum* apart from being a natural vector for *P*. *knowlesi* [17].

#### Other organismal system-related functions

Apart from immune system-related functions, there were unique genes from the KEGG organismal systems annotated to the sensory system as well. Six *An*. *cracens* unique genes were annotated to phototransduction (anopheles.cracens_GLEAN_10008160, 10008159, 10013726, 10000620, 10023751, and 10004812). Another unique gene (anopheles.cracens_GLEAN_10024588) was annotated to environment adaptation and circadian rhythm. Similarly in *An*. *maculatus*, there was one gene annotated to phototransduction (anopheles.maculatus_GLEAN_10017051) and four of its unique genes were annotated to environment adaptation and circadian rhythm (anopheles.maculatus_GLEAN_10023357, 10027410, 10026232, and 10008259). These genes are likely to be involved in shaping the living habitat and exophilic feeding habit, and feeding period of *An*. *cracens* and *An*. *maculatus* after sunset. Meanwhile, the moon phase has been reported to affect the oviposition rate and host seeking behavior of a few *Anopheles* species [95, 96]. The effects of moon phase on feeding behavior and oviposition of *An*. *cracens* and *An*. *maculatus* are yet to be reported. We do not know whether these genes are responsible for such phenomena (if any). Nevertheless, it would be interesting to look deeper into this aspect for future studies.

The data gathered from this study suggest that multiple genetic factors are involved in shaping the feeding behavior of *Anopheles* mosquitoes, encompassing odorant reception, circadian rhythm, light and temperature sensitivity. The immune system of the mosquitoes sets a “firewall” against invading foreign particles such as parasites. However, depending on parasite inoculation (feeding) frequency and relative amount of parasites introduced, the mosquito immunity protection may be bleached, making the mosquito susceptible to the infection, hence becoming a vector. Therefore, the susceptibility of *An*. *cracens* and *An*. *maculatus* to *Plasmodium* spp. in natural and laboratory settings may differ. Because of constrain of time and resources, we were not able to perform downstream experiments involving gene manipulations. Nevertheless, our findings have highlighted a number of important and interesting features for future studies.

## Conclusions

The draft genomes of *An*. *cracens* and *An*. *maculatus* presented here add to the anopheline vector genome database. The *An*. *maculatus* draft genome reported by this study may represent a different geographical race from the already published Malaysian *An*. *maculatus* genome. Comparisons revealed similarities and differences between *An*. *cracens* and *An*. *maculatus* at genomic level, which may explain their similarities and differences in feeding behaviors and susceptibility to human malaria parasites and simian malaria parasites, under natural and laboratory conditions.

## Supporting Information

S1 Table Statistic of pre-filter data. (XLSX)

S2 Table Statistic of post-filter data. (XLSX)

S3 Table Transposable elements (TEs) content in the assembled *An*. *cracens* and *An*. *maculatus* genomes. (XLSX)

S4 Table General statistics of predicted protein-coding genes. (XLSX)

S5 Table Functional annotation statistics. (XLSX)

S6 Table Non-coding RNA genes in genomes of *An*. *cracens* and *An*. *maculatus*. (XLSX)

S7 Table Statistics of genome-scale comparison of different Diptera members. (XLSX)

S8 Table Positively selected genes (PSGs) of *An*. *cracens* and *An*. *maculatus*. (XLSX)

S9 Table InterProScan annotations for PSGs of *An*. *cracens*. (XLSX)

S10 Table InterProScan annotations for PSGs of *An*. *maculatus*. (XLSX)

S11 Table *An*. *cracens* OR genes distribution. (XLSX)

S12 Table *An*. *maculatus* OR genes distribution. (XLSX)

S13 Table *An*. *cracens* OR genes that are not shared with *An*. *maculatus*. (XLSX)

S14 Table *An*. *maculatus* OR genes that are not shared with *An*. *cracens*. (XLSX)

S15 Table Unique *An*. *cracens* genes mapped to KEGG pathways. (XLSX)

S16 Table Unique *An*. *maculatus* genes mapped to KEGG pathways. (XLSX)

S17 Table (A) Global statistics of *An*. *maculatus* genome assembled in this study, which was sequenced with 93.21x coverage; (B) *An*. *maculatus* genome assembled by previous study (Neafsey *et al*., 2015), which was sequenced with 25x genome coverage (BioProject code PRJNA67221, BioSample code SAMN01087922).
(XLSX)

S18 Table Alignment of two *An*. *maculatus* genomes, with the genome assembled from this study as the target genome and the published genome as query genome. (XLSX)

S19 Table *K-mer* analysis of the published *An*. *maculatus* genome (BioProject code PRJNA67221, BioSample code SAMN01087922). (XLSX)
